# Unified metagenomic method for rapid detection of microorganisms in clinical samples

**DOI:** 10.1038/s43856-024-00554-3

**Published:** 2024-07-07

**Authors:** Adela Alcolea-Medina, Christopher Alder, Luke B. Snell, Themoula Charalampous, Alp Aydin, Gaia Nebbia, Tom Williams, Simon Goldenberg, Sam Douthwaite, Rahul Batra, Penelope R. Cliff, Hannah Mischo, Stuart Neil, Mark Wilks, Jonathan D. Edgeworth

**Affiliations:** 1 Infection Sciences, Synnovis, London, UK; 2 Center for Clinical Infection and Diagnostics Research, Guys’ and St. Thomas’ NHS Foundation Trust, London, UK; 3 Department of Infectious Diseases, King’s College London, London, UK; 4 Quadram Institute Bioscience, Norwich, UK; 5 Department of Infectious Diseases, Guys’ and St Thomas’ NHS Foundation Trust, London, UK; 6 Queen Mary, University of London, London, UK

**Keywords:** Clinical microbiology, Metagenomics

## Abstract

**Background:**

Clinical metagenomics involves the genomic sequencing of all microorganisms in clinical samples ideally after depletion of human DNA to increase sensitivity and reduce turnaround times. Current human DNA depletion methods preferentially preserve either DNA or RNA containing microbes, but not both simultaneously. Here we describe and present data using a practical and rapid mechanical host-depletion method allowing simultaneous detection of RNA and DNA microorganisms linked with nanopore sequencing.

**Methods:**

The human cells from respiratory samples are lysed mechanically using 1.4 mm zirconium-silicate spheres and the human DNA is depleted using a nonspecific endonuclease. The RNA is converted to dsDNA to allow the simultaneous sequencing of DNA and RNA.

**Results:**

The method decreases human DNA concentration by a median of eight Ct values while detecting a broad range of RNA & DNA viruses, bacteria, including atypical pathogens (*Legionella*, *Chlamydia*, *Mycoplasma*) and fungi (*Candida, Pneumocystis, Aspergillus*). The first automated reports are generated after 30 min sequencing from a 7 h end-to-end workflow. Sensitivity and specificity for bacterial detection are 90% and 100%, respectively, and viral detection are 92% and 100% after 2 h of sequencing. Prospective validation on 33 consecutive lower respiratory tract samples from ventilated patients with suspected pneumonia shows 60% concordance with routine testing, detection of additional pathogens in 21% of samples and pathogen genomic assembly achieve for 42% of viruses and 33% of bacteria.

**Conclusions:**

Although further workflow refinement and validation on samples containing a broader range of pathogens is required, it holds promise as a clinically deployable workflow suitable for evaluation in routine microbiology laboratories.

## Plain language summary

Metagenomics is the analysis of genetic material from microbes such as bacteria and viruses in a sample. There are limitations with existing metagenomics methods, such as not being able to detect the full range of microbes present in a sample. This paper introduces an approach that identifies multiple types of microbes. This is accomplished through the mechanical disruption of human cells, which allows for an effective depletion of human genetic material. Our method demonstrates encouraging preliminary results within a 7 h process, achieving good sensitivity for the detection of bacteria and viruses. We demonstrate the identification of relevant microbes in samples from patients with respiratory infections. This technique holds promise for adoption in clinical settings, potentially enhancing our ability to diagnose respiratory infections quickly.

## Abstract (web summary)

Alcolea-Medina et al. develop a rapid, unified metagenomics method for the detection of bacteria, fungi, and viruses from clinical respiratory samples. This method reduces human DNA content, allowing efficient pathogen detection and possibility of same-day reporting to meet requirements of clinical implementation.

## Introduction

Clinical metagenomics has the potential to revolutionise the initial management of acute infections by rapidly identifying and characterising all pathogenic microorganisms in clinical samples within a few hours^1^. The potential of metagenomics is particularly relevant for lower respiratory tract infections (LRTI) that are the 4th biggest cause of mortality globally^2^ and the most common cause of death from sepsis^3^ and can be caused by the broadest range of known and emerging bacterial, fungal and viral pathogens^4^. The current diagnostic approach combines culture and targeted multiplex PCR for viruses and atypical respiratory pathogens, supplemented by antigen detection and other tests. These multiple assays provide staggered and incomplete information, which causes residual uncertainty for diagnosis and management particularly during the first few hours and days. Operationally, samples are often processed on different benches or in different laboratories or require sending away to reference laboratories if the presence of rarer and fastidious pathogens is suspected or for typing which further delays receipt of all required information^5^.

Different human DNA depletion methods have been developed using chaotropic agents such as saponin, or differential centrifugation to physically separate microorganisms from host cells^6,7^. To our knowledge, none of these depletion methods is considered sufficiently efficient at preserving all these different microorganisms to a level required for clinical testing, given fundamental differences in their physicochemical properties and abundance in human samples^8,9^. Splitting samples and detecting viruses in the supernatant and bacteria or fungi in the deposit before mixing back at the molecular stage is one solution^8,10^, but this adds time, cost and complexity which is not ideal in a clinical laboratory.

A key challenge is developing a method with the necessary scientific capabilities that remains technically and cost-effectively deployable in a routine service laboratory with appropriate quality controls, generating results that meet clinical needs by providing actionable reports within hours of sample receipt. We recently evaluated a 7 h respiratory metagenomic research workflow into a pilot study, using the saponin method, service setting after evaluation on clinical samples, incorporation of quality controls and generation of standardised clinical reports^8,9^. The saponin method used preserves bacteria, some fungi and some DNA viruses, but not RNA viruses that are an important target for a respiratory metagenomic assay^11^.

Here, we provide detailed technical data on the development of a unified rapid mechanical human DNA depletion method involving centrifugation and bead beating prior to nucleic acid extraction. When combined with reverse transcription, PCR-based cDNA amplification and nanopore sequencing, the workflow generates reports within 7 h, and detects bacteria, fungi and DNA and RNA viruses in both upper and lower respiratory samples. We also present preliminary comparative performance data when applied to respiratory samples from a cohort of adult and paediatric patients admitted with severe community-acquired pneumonia over a winter season.

## Methods

### Upper and lower respiratory tract samples

Surplus samples were retrieved from the clinical microbiology laboratory after routine testing was completed. These samples were anonymised before being submitted to the research team along with their routine test results (Ethical approval: North West Preston REC reference 18/NW/0584). The Institutional Review Board waived the need for informed consent because the samples were routinely collected and de-identified prior to submission to the research laboratory. Samples were selected based on reported detection of viruses and bacteria by the clinical laboratory to facilitate rapid method evaluation.

Fifty respiratory samples (42 combined nose and throat swabs (NTS) in viral transport medium (VTM), five bronchoalveolar lavages (BAL) and three sputa) were tested to evaluate viral performance characteristics, sensitivity and specificity. Because of the potential for viral loss during storage in the routine laboratory, the research team first repeated viral PCR on the un-depleted aliquot to confirm clinically reported results and allow comparison with the metagenomic method. Metagenomic sequence results were compared with the VIASURE Respiratory Panel III Real-Time PCR Detection Kit (Certest Biotec^TM^), which detects Influenza A (Flu A), Influenza B (Flu B), Human Respiratory Syncytial Virus (RSV), Parainfluenza 1 (PIV-1), Parainfluenza 2 (PIV-2), Parainfluenza 3 (PIV-3), Parainfluenza 4 (PIV-4), human Adenovirus (AdV), Metapneumovirus (MPV), Bocavirus (BoV), human rhinovirus (HRV), human enterovirus (HEV), Coronavirus (CoV) 229E, NL63, OC43, HKU1 strains; *Chlamydophila pneumoniae*, *Mycoplasma pneumoniae*, *Legionella pneumophila*, *Haemophilus influenzae*, *Streptococcus pneumoniae* and *Moraxella catarrhalis*. 33 samples were PCR positive for one or more viruses and 17 PCR negative for all PCR-detected viral pathogens.

To determine performance characteristics for bacterial detection, 48 lower tract respiratory samples (20 BAL, 13 pleural fluids (PF), three non-directed bronchoalveolar lavages (NBL), 11 sputa and one endotracheal aspirate (ETT)) were analysed. Metagenomic results were compared with semi-quantitative culture and any other tests performed by the clinical laboratory including pneumococcal and *Legionella* urinary antigen (Binax NOW, Abbott^TM^) or 16S rRNA gene sequencing, which is performed by an external laboratory^12^.

Thirty-three lower respiratory tract samples (33/48) were reported culture-positive for putative or likely respiratory bacterial pathogens and fifteen samples (15/48) as ‘no organisms detected’ or ‘no significant organism’. Five samples grew *Candida* and one sample was PCR positive for *P. jirovencii*. To check concordance for the detection of *Aspergillus fumigatus*, when this was detected only by sequencing, a targeted PCR was performed as previously described^12^.

### Human DNA depletion and microbial RNA and DNA extraction

Samples were first centrifuged at 1200*g* for 10 min to pellet human cells, then 500 µL of supernatant was subjected to bead-beating in 2 mL of Lysing Matrix D (MP biomedical^TM^) for 3 min at 50 oscillations/s in the TissueLyser LT (Qiagen^TM^) to lyse human cells. In total, 200 µL was then transferred to an 1.5 mL Eppendorf tube with 10 µl of HL-SAN nuclease (ArcticZymes Technologies^TM^) without buffer and incubated at 37 °C for 10 min at 1000 rpm on a thermomixer (Eppendorf^TM^) to digest released human nucleic acid. HL-SAN nuclease digests RNA at roughly 10-fold less efficiency than DNA. Samples containing preserved intact microorganisms were then extracted to release DNA and RNA from bacteria, viruses and fungi in the MagNA Pure 24 System (Roche^TM^) using total NA isolation kit 1.1 with pre-set bronchoalveolar lavage sample parameters at 200 μL input volume and 50 μl elution volume. Fast pathogen 200 1.1 was used for processing <8 samples and Pathogen 200 3.2 for ≥8 samples.

### cDNA and double-strand DNA synthesis

For cDNA synthesis, 4 µL of LunaScript® RT SuperMix Kit (New England Biolabs^TM^) was added to 16 µL of nucleic acid extract and incubated following the manufacturer’s conditions. Sequenase version 2.0 (Thermo Fisher^TM^) was used for double-strand DNA synthesis, with 2 μL of 5× Sequenase buffer, 0.9 μL of Sequenase dilution buffer, 0.6 μL of Sequenase and 7.7 μl of nuclease-free water (Thermo Scientific^TM^) added to 20 µL of template from the previous reaction, then incubated at 37 °C for 8 min. Samples were cleaned using 45 µL of AMPure XP beads added to the 31.2 µL obtained from the dsDNA synthesis step and incubated for 5 min at room temperature in a new 1.5 mL Eppendorf tube. The Eppendorf tube was then placed in a magnetic rack for 2 min before removal of the supernatant, and the pellet  was washed twice with 70% ethanol followed by elution in 10 μL of nuclease-free water (Thermo Scientific^TM^).

### Library preparation and sequencing

DNA was prepared for sequencing using the Rapid PCR barcoding kit (SQK-RPB004—Oxford Nanopore Technologies (ONT)) following the manufacturer’s recommended conditions apart from increasing PCR cycles to 30. Samples were sequenced using flowcells (R9.4.1) on a GridION platform (ONT), multiplexing between 3 and 10 samples per flowcell. Raw nanopore reads were demultiplexed and base-called using Guppy (version 6.1.5) within MinKNOW (version 22.05.7), filtering reads with a *q-*score <7 and length <200 base pairs (bp). The parameter ‘barcode-at-both-ends’ was used during demultiplexing to mitigate any barcode misclassification. A “no template negative control” (nuclease-free water, Thermofisher^TM^) was added to each run alongside the samples tested.

### Assessing the efficiency of human DNA depletion and microbial recovery

Human DNA depletion was assessed, using a targeted PCR assay targeting human RNA polymerase 2, in 29 samples. An aliquot of 200 µL was taken before the human DNA depletion process, omitting the centrifugation, bead-beaten and HL-SAN treatment; another aliquot was depleted, and both were extracted as previously detailed. The  impact  of the centrifugation step alone was assessed on 3 paired VTM aliquots (two from patient NT swabs and one a spiked sample) with one aliquot having the initial centrifugation step omitted. One NT swab with *P. aeruginosa* reported by culture was spiked with Adenovirus, SARS-CoV-2 and PIV3 (Zeptometrix®), one NT swab had seasonal coronavirus reported by routine testing and one sterile VMT sample was spiked with NATtrol™ Respiratory Panel 2.1 (RP2.1), Zeptometrix®) and a 0.5 McFarland standard of clinical isolates of *P. multocida*, S. *aureus*, *C. albicans* and *H. influenzae*.

Impact of the human DNA depletion process on viral recovery was assessed by analysing the 29 paired pre and post depletion clinical samples evaluated for human DNA depletion, and three paired pre and post depletion negative BAL samples spiked with NATtrol™ Respiratory Panel 2.1 (RP2.1) (Zeptometrix®). Viral quantification in these paired samples was assessed using the VIASURE Respiratory Panel III Real-Time PCR Detection Kit (Certest Biotec^TM^) (Supplementary Data 14).

Impact of the human DNA depletion process on bacterial and *Candida spp* recovery was assessed by spiking BAL samples that were negative by routine testing, with *S. aureus* (NCTC 6571), *K. pneumoniae* (NCTC 13368), *S. pneumoniae* (NCTC 12977), *H. influenzae* (NCTC 13381) or *C. albicans* (NCPF 3178) strains obtained from UK Health Security Agency. Targeted PCRs against the spiked microorganisms was performed on pre and post-depletion aliquots (Supplementary Data 13)^11,13,14^.

Paired aliquots from three BAL samples taken before and after the human DNA depletion process, were to determine how the how human DNA depletion was increasing the sequencing sensitivity. One was reported by routine testing as ‘commensals’ and the others two were reported high and light growth of *P. aeruginosa*.

A 16S rRNA gene qPCR assay targeting the V3-V4 fragment^15^ was performed on an aliquot from ten randomly selected samples taken before and after the combined RT and dsDNA synthesis steps to determine their impact on bacterial DNA recovery.

### Prospective validation study

Routinely collected samples received from ventilated patients with suspected pneumonia in the paediatric and adult ICU were identified for sequencing after daily review by the service laboratory team between December 2022 and January 2023. Samples were anonymised and matched with results from all routinely requested test results. 33 samples were retrieved comprising 22 BAL/NBL, 6 pleural fluids, 4 sputa and one ETT, representing 70% of all samples meeting inclusion criteria. Concordance with results of routinely requested tests was compared with sequence reports after 30-min, 2-h and 24-h of sequencing. Pathogen genome construction was attempted from 24-h sequence data. Results were not reported to clinicians as the samples were anonymised for the study.

### Viral, bacterial and fungal detection in serial dilutions of spiked samples

Serial dilution experiments were performed on BAL samples with no pathogens reported by the routine laboratory and containing either low (Ct ≥ 26 Ct) or high (Ct ≤ 14) background commensal bacterial flora determined by qPCR for the 16S rRNA gene. Targeted PCR was used to confirm the absence of spiked organisms from the primary sample.

*S. aureus* NCTC 6571, *K. pneumoniae* NCTC 13368 or *C. albicans* ATCC 10231 were spiked in triplicate at concentrations of 10^5^, 10^4^ and 10^3^ CFU/mL. Influenza A1H1 (Zeptometrix^TM^) and Human Herpes Virus Type 6 (Zeptometrix^TM^) were spiked in duplicate into BAL and NTS-VTM samples at between 250 copies/ul to 10 copies/ul. All dilutions were assessed for spiked viruses by targeted PCR, using VIASURE Flu A + B Real-Time PCR Detection Kit and VIASURE Human Herpes Virus 6, 7 and 8 Real-Time PCR Detection Kit.

### Bioinformatic analysis

#### Metagenomics analysis

Fastq read files were batched according to the reporting sequencing run times tested (30 min, 2 h or 24 h). Before downstream analysis, human reads were removed with alignment against human reference (GCA_000001405.15) using minimap2 (v2.18r-1015)^16^. Microbial reads were first classified for viral species using Centrifuge (version 1.04) against an NCBI RefSeq viral database^17^. Only reads with a Centrifuge score > 500 were considered for further analysis, except for reads classified as Enterovirus/Rhinovirus, which were considered with a Centrifuge score > 100. Reads mapping to multiple viruses were assigned the closest taxonomic ranking they shared. Virus classifications were filtered and reported only if they were from clinically relevant genera from a list of predefined pathogens (Supplementary Data 1).

Using the training dataset, classification scores used for the presence or absence of pathogenic organisms were established to mitigate the misclassification of sequencing reads and the number of false calls. For the bacterial and fungal classification, previously described thresholds^12^ were tested and found to translate optimally for this workflow. Thresholds for classification scores of viral reads were assessed for several classification scores (100, 250, 500 and 1000).

Reads unclassified within the viral database were extracted and classified using Centrifuge against a custom bacterial and fungi database^12,16^. Classified reads with a centrifuge score of ≥8000 were considered in further analysis. Reads matching to multiple species were aligned against their respective reference assembly using minimap2 and BLAST identity was calculated to determine best species assignment previously described^12^. Bacterial species were reported if they represented ≥10 reads, ≥1% of total bacterial classified reads, and were either in a list of predefined pathogens or species of oral flora. Reporting thresholds for *Candida spp* and *Aspergillus spp* were set at >5 reads based on previously determined thresholds^12^. Other fungal pathogens were considered if ≥2 reads were present in the dataset.

#### Viral and bacterial assembly

For viruses and bacteria with more than 90 and 10,000 reads respectively, reference-based assembly was performed. Sequence reads classified as the target organism were extracted and mapped to RefSeq reference assemblies using minimap2. Variant calling was performed using Medaka (version 1.7.2). Masking beds were created for various depths 10×,15×,20×,30× and draft consensus sequences were created using these masking beds with samtools/bcftools (version 1.15.1)^17,18^. Metaquast (version 5.2.0) was used to assess genome quality to identify the closest related species/strain to draft consensus sequences, using the NCBI viral Blast database for viral assemblies, and the SILVA 16 Sv Blast database for bacterial assemblies^19^.

#### AMR analysis

The first 2 h of non-human sequencing data were analysed for resistance determinants using abricate (version 1.0.1) (https://github.com/tseemann/abricate) and the CARD database. Genes associated with relevant pathogens within the dataset were extracted using Scagaire (version 0.0.4) (https://github.com/quadram-institute-bioscience/scagaire). Genes identified were considered if the alignment coverage was >90% and identified ≥2 reads as previously described^12^. Genotypic determinants were only considered for the following organisms and antimicrobials: extended-spectrum beta-lactamase and carbapenamases for Enterobacterales, mecA for *S. aureus*, and vanA for *E. faecalis* as previously described^12^.

For viruses, SNP distance was determined with snp-dists (v0.8.2) after alignment with mafft v7.490 with default parameters. Lineage and clades for SARS-CoV-2, RSV and influenza were determined using NextClade 2.14.1 (https://clades.nextstrain.org/https://clades.nextstrain.org/). Plasmid analysis for bacteria was performed using PlasmidFinder 2.0.1 (ref: https://www.ncbi.nlm.nih.gov/pmc/articles/PMC4068535/https://www.ncbi.nlm.nih.gov/pmc/articles/PMC4068535/) with default parameters. A complete reference genome for *Streptococcus pyogenes* (ATCC 19615) (NZ_CP008926.1) was retrieved from RefSeq to be used to generate consensus sequences. Reads were extracted from 2 samples of metagenomic sequencing runs mapping to *S.pyogenes*, and aligned to the reference assembly. Consensus sequences were generated using bcftools v1.10 following and regions coverage below 10x were masked. SNP-sites v2.5.1 was used to identify SNP differences common between the samples and a SNP matrix was generated using SNP-dists v0.8.2. Only genomic positions with sufficient depth between the two samples were considered in the analysis. In total, 1.19 Mb of the 1.84 Mb genome was covered in both samples at 10x depth. Of these positions, a total of 12,621 SNPs were identified between the two samples.

### Clinical report generation and reporting thresholds

Organism detections were listed in automated reports generated after 30 min, 2 h and 24 h sequencing. The 24 h data were also used for genome assembly to assess genome recovery.

The optimal cut-off for reporting Gram-positive and Gram-negative bacteria that are not obligate pathogens and that are currently detected by semi-quantitative culture was assessed using ROC analysis at 0.5%, 1%, 2%, 5% and 10% abundance after 0.5, 2 and 24 h sequencing. No lower abundance limit was set for obligate pathogens, atypical respiratory bacteria and viruses for which any detection by the routine laboratory is generally considered significant. ROC analysis for RNA viruses was performed using absolute read number starting at a single read. ROC analysis was not performed for DNA viruses given there were few examples, and these are not routinely tested or considered pathogens in respiratory samples by the diagnostic laboratory. A reporting threshold for *Candida spp* was pragmatically set at least >5 reads based on earlier results. Figures displaying ROC analysis were made in StataMP 17.0 (StataCorp LLC, USA).

Organisms detected in the negative control from that run were removed from sample reports whether present below or above the threshold.

### Definitions

True positive results: metagenomic sequencing results were concordant with the microbial detections provided by the clinical laboratory and the Viasure kit results. True negative results: no reportable organisms were identified by metagenomics or by the clinical laboratory. False-negative results were considered when the clinical laboratory reported bacteria or viruses that were not found by sequencing. Additional detection: The sequencing method detected additional pathogens in samples that had already been reported as positive for the detection of pathogens by both methods.

### Reporting summary

Further information on research design is available in the Nature Portfolio Reporting Summary linked to this article.

## Results

### Human DNA depletion and microbial preservation and recovery

**Fig. 1:**
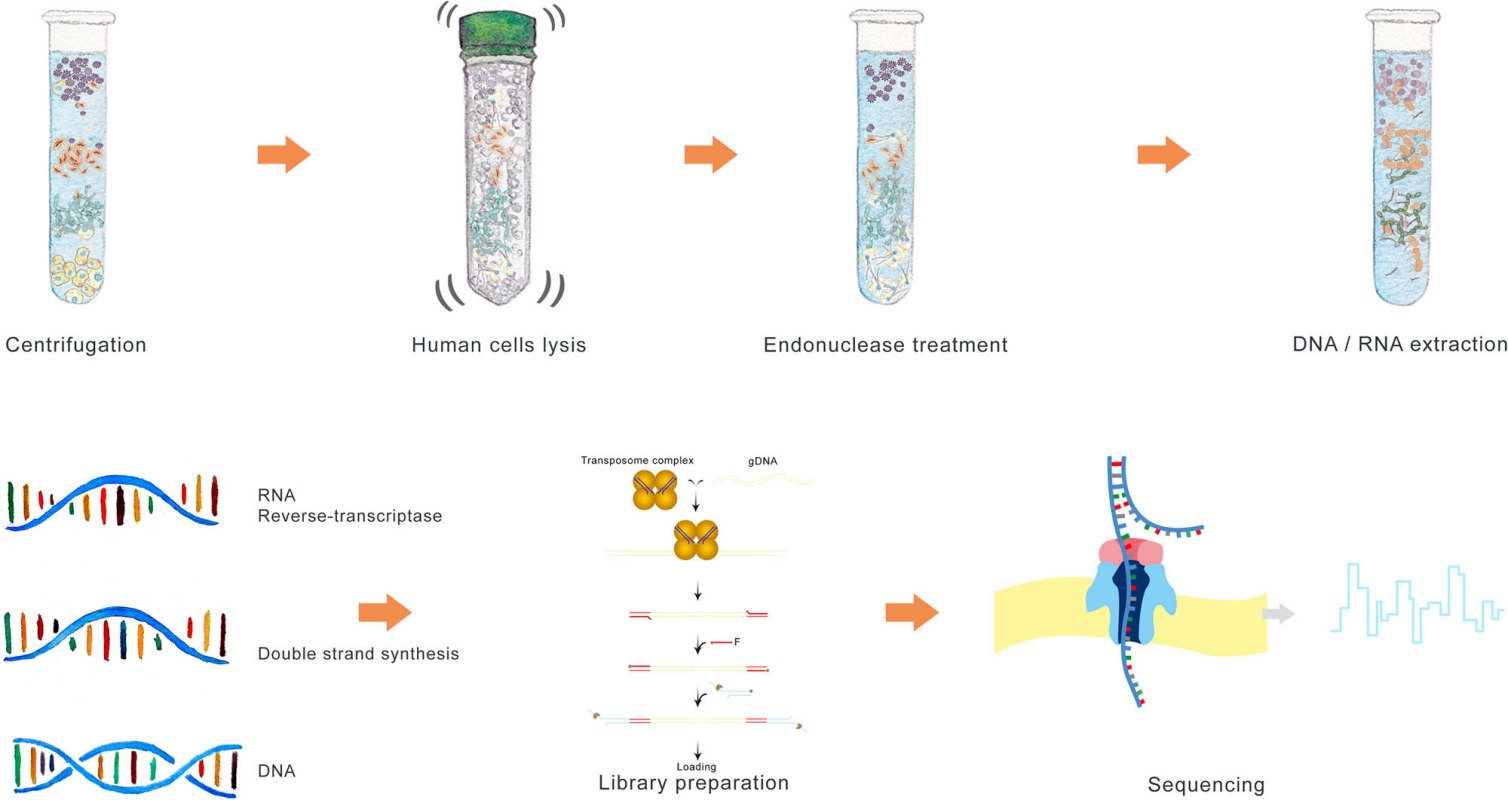
Metagenomics workflow. The first step involves spinning the sample. Most of the human cells settle at the bottom, allowing for the collection of supernatant containing mainly microorganisms. The remaining human cells in the supernatant are lysed using mechanical disruption to release the DNA. A nonspecific endonuclease is added to digest the cell-free DNA and RNA present in the supernatant. DNA and RNA-containing microorganisms are extracted. After the extraction, RNA is converted into complementary DNA (cDNA) using the enzyme reverse transcriptase. The single-stranded cDNA is used as a template to synthesise a complementary strand, forming double-stranded DNA. The library preparation is performed using SQK-RPB004—Oxford Nanopore Technologies (ONT). Elements of this Figure are adapted with permission from ONT.

Human DNA was quantified in 29 NT samples with and without host depletion steps of the protocol (Fig. 1) which showed a median depletion of seven cycle thresholds (IQR 5–10). Median human DNA depletion from 29 lower respiratory tract samples was ten cycle thresholds (IQR 4–12). Overall, the human DNA depletion across upper and lower respiratory tract samples was eight (IQR 4–12), representing an approximately 256-fold reduction (Supplementary Data 2 and 3).

Aliquots of three BAL samples were sequenced in parallel with and without host depletion to compare the impact on human and microbial sequence reads. Each sample showed decreases in human reads and increases in microbial reads, although across a wide range of absolute numbers and percentages for each measure in the three samples (Table 1). When the aliquots of three samples were also sequenced in parallel with and without the initial centrifugation step of the host-depletion process, the human DNA decreased in both samples increasing the detection and sequencing of the RNA viruses. Nevertheless, the quantity of bacterial reads exhibited wide variability (Table 2).

**Table 1:** Comparison of human and microbial DNA recovery with and without host depletion

Sample number	Sample type	Routinely reported results	Depletion	Total number of read	Human Reads	Microbial reads	Target organisms
1	BAL	*P. aeruginosa* (M)	No	378,366	373,548 (99%)	3377 (0.9%)	2878 (0.8%)
			Yes	213,695	41,635 (15%)	152,418 (71%)	140,544 (66%)
2	BAL	Upper respiratory tract flora	No	145,961	145,926 (99.9%)	2	2
			Yes	16,057	3594 (22%)	7069 (44%)	7069 (44%)
3	BAL	*P. aeruginosa* (S)	No	36,343	35,778 (98%)	368 (1%)	187 (0.5%)
			Yes	24,145	21,715 (90%)	1224 (5%)	579 (2.4%)

**Table 2:** Comparison of human and microbial DNA recovery with and without initial centrifugation

Sample number	Sample type	Reported (spiked)	Centrifugation	Total number of read	Human Reads	Microbial reads	Number of reads of the target organisms
1	NT	*P.aeruginosa* (Adenovirus SARS-CoV-2) Herpesvirus 7 (PIV3)	Yes	620,452	2332 (0.37%)	618,130 (99.6%)	609,514 (98%) 2,684 446 34 15
			No	539,843	22,555 (0.042%)	517,288 (96%)	250,999 (46%) 47 3 2 0
2	Spiked sterile VTM	(*P. multocida* *S. aureus* *C. albicans* *H. influenzae* Adenovirus *M. pneumoniae* *C. pneumoniae* PIV1 PIV3 PIV4 FluA Rhinovirus A Metapneumovirus Coronavirus OC43 Coronavirus 229E Coronavirus NL63 SarsCov2 RSV)	Yes	2,054,974	N/A	2,050,603 (99%)	505,388 (24.5%) 1,147,990 (56%) 1787 (0.1%) 1263 (0.06%) 9531 175 60 6 16 4 6 2 1 2 3 1 1 0
			No	958,291	N/A	957,640 (99%)	813,470 (85%) 117,570 (12%) 6570 (0.68%) 952 (0.1%) 134 3 1 0 0 0 0 0 0 0 0 0 0 0
3	NT	Human CoV HKU1	Yes	222,732	17,859 (8%)	204,873 (92%)	191
			No	301,165	36,644 (12%)	264,521 (88%)	4

Reductions of DNA and RNA viral nucleic acid due to the human DNA depletion method were assessed using targeted viral PCR on 17 known viral PCR-positive NT swabs and showed a median reduction of three cycles thresholds (IQR 2–7). However, when the same comparison was performed on viral PCR negative BAL samples spiked with the positive control panel containing whole viruses the median decrease was only 1.8 Ct values (IQR 1–2) (Supplementary Data 4). Reductions in the level of detection of four representative bacterial (2 Gram-positive and 2 Gram-negative) and Candida albicans nucleic acid by the human DNA depletion were assessed in three spiked BAL samples and showed only small reductions in cycle threshold (median Ct change of 0) (Supplementary Data 5).

The impact of reverse transcription and dsDNA synthesis steps on bacterial DNA recovery was assessed by measuring 16S rRNA PCR in parallel on un-depleted and human DNA-depleted aliquots and an aliquot recovered post dsDNA synthesis (*n* = 10). 16S rRNA PCR Ct values consistently increased after human DNA depletion but then reduced after the RT-dsDNA steps (range 1–9) (Supplementary Data 6).

### Serial dilution experiments detecting viruses, bacteria and yeast

Serial dilutions were performed on a representative RNA virus (influenza A) and DNA virus (HHV-6) in lower respiratory tract samples (BALs) and nasal throat swabs (NTS), in presence of both high and low microbial commensal background. Influenza A was detected at 70 copies/µL and HHV-6 at 10 copies/µl, both regardless of the bacterial microbial commensal background quantity (Supplementary Data 7). For bacteria, *S. aureus* was detected at 10^3^ CFU/mL in BALs with low commensal background and 10^5^ CFU/mL with high commensal background, and *K. pneumoniae* was detected at 10^3^ CFU/mL in both high and low commensal samples (Supplementary Data 8). For *C. albicans* the lowest detection was at 10^3^ CFU/mL in both high and low commensal backgrounds (Supplementary Data 9).

### Development data set to determine representative viral performance characteristics

**Fig. 2:**
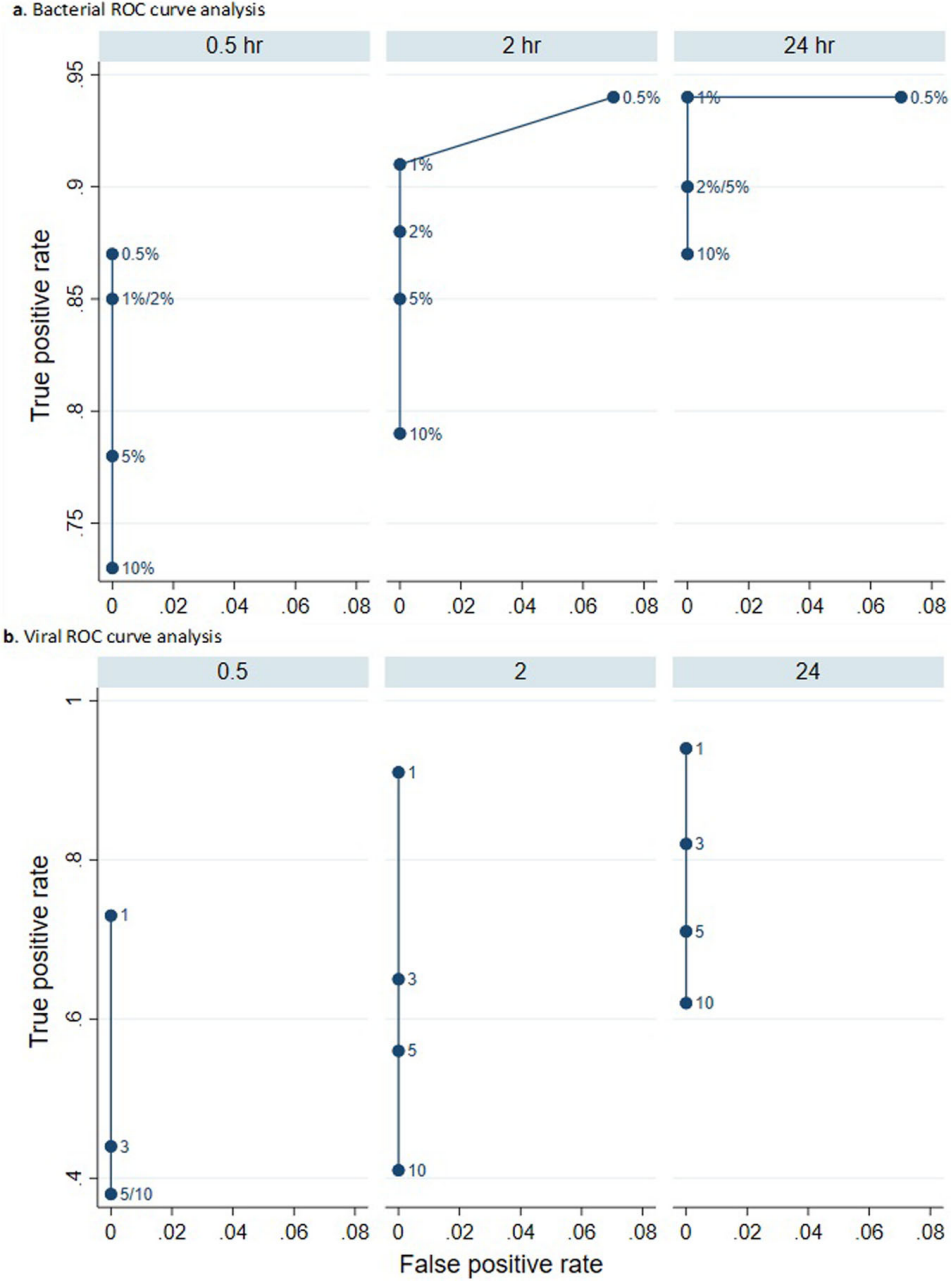
Bacterial and Viral ROC curve analysis. **a** ROC curves were constructed to establish the bacterial and viral reporting thresholds. True positive rate (sensitivity) against the false positive rate (1-specificity) for bacterial detection at different bacterial abundance thresholds (0.5%, 1%, 2%, 5%, and 10%) after 0.5 h, 2 h, and 24 h of sequencing. The *y*-axis represents the true positive rate, indicating how the metagenomics method detects actual cases of bacterial presence as confirmed by standard culture and molecular assays. The *x*-axis represents the false positive rate, reflecting the proportion of false positives among the negatives identified by the clinical assays. **b** This ROC curve focuses on viral read counts (1, 3, 5, and 10) after 0.5 h, 2 h, and 24 h of sequencing. Again, the *y*-axis shows the true positive rate, measuring how accurately the metagenomics detects viruses relative to a standard multiplex PCR assay. The *x*-axis shows the false positive rate. Points represent thresholds for the number of viral reads necessary to report a positive result.

Sensitivity and specificity for detecting clinically reported viruses in 50 respiratory samples were 77% and 100% after 30 min, 92% and 100% after 2 h, and 94% and 100% after 24 h sequencing, respectively (Table 3). There were no false positive metagenomic viral detections in viral PCR negative samples, so the threshold for reporting was set at 1 read to maximise sensitivity (Fig. 2). Increase in sensitivity after 2 h was due to additional detection of five viruses (samples 9, 13, 14, 41 and 39) and after 24 h from additional detection of a parainfluenza virus 3 (sample 67) identified by PCR with a Ct value of 30.

**Table 3:** Viral sensitivity and specificity after 0.5 min, 2 h and 24 h sequencing

Number of reads	30 min sequencing		2 h sequencing		24 h sequencing	
	Sensitivity (%)	Specificity (%)	Sensitivity (%)	Specificity (%)	Sensitivity (%)	Specificity (%)
1	77	100	92	100	94	100
3	50	100	72	100	83	100
5	38	100	63	100	75	100
10	25	100	44	100	66	100

Clinically reported viruses were missed in two NT samples, a parainfluenza virus 3 (sample 1) and an enterovirus (sample 42), both with Ct > 30 by PCR (Table 4). Influenza virus C was identified in sample 50, which is not targeted by the multiplex PCR (Table 5). Clinically relevant respiratory viruses were also detected in three sputum samples that had not had viral PCR tests requested by clinicians: influenza A virus, SARS-CoV-2 and enterovirus A virus (samples number 36, 37 and 49) (Supplementary Data 10). All were confirmed by Viasure PCR.

**Table 4:** Missed detection by metagenomics pipeline in polymicrobial samples

Sample number	Sample type	Routine testing detections (Ct values for viral detections or bacterial/fungal culture growth)		2 h of sequencing			
				Microorganisms sequenced		Number of microbial reads	Percentage of bacterial reads
1	NTS	SARS-CoV-2 (Ct 20/22) PIV3 (Ct 29)		*SARS CoV-2* *Human gammaherpesvirus 4*		2878 1188	57.514 23.741
79	BAL	*B.cepacia (L)*		*B. cepacia*		53	0.671
73	Sputum	*E. faecalis (H)* *E. coli (S)* *C. albicans (S)* B-glucan positive		*E. faecalis* *A. fumigatus* *C. albicans* *P. jirovecii*		119 18 4 1	1.277 N/A N/A N/A
88	*BAL*	*A. baumanii (H)* *P. aeruginosa (H)* *E. meningoseptica (L)*		*P. aeruginosa* *A. baumannii* *S. maltophilia*		483 412 123	4.178 3.564 1.064
42	BAL	Enterovirus/Rhinovirus (Ct 35) Seasonal coronavirus (Ct 30)		*P.melaninogenica* *S. salivarius* *S. sp. FDAARGOS_192* *S. equinus* *N. mucosa* *V. parvula* *R. mucilaginosa* *P. jejuni* *P. salivae* *P. scopos* *S. odontolytica* *S. viridans* *G. sanguinis* *Human coronavirus 229E*		1465 441 325 150 123 118 105 104 84 69 67 65 38 30	40.481 12.186 8.98 4.145 3.399 3.261 2.901 2.874 2.321 1.907 1.851 1.796 1.05 N/A

*H* high growth, *L* light growth, *S* scanty growth.

**Table 5:** Additional potential pathogens detected

Sample number	Sample type	Routine testing detections	Sequencing results after 2 h		
			*Microorganisms (Ct values)*	Number of microbial reads	Percentage of bacterial reads
51	BAL	Agar culture negative *S. pneumoniae* urine Ag positive	*S. pneumoniae*	13	26.531
11	Sputum	Commensals	*R. mucilaginosa* *S. mitis* *S. pneumoniae* *S. oralis* SARSCov2 (Ct 20) *P. histicola*	29765 1014 835 549 413 395	87.446 2.979 2.453 1.613 N/A 1.16
20	BAL	Agar culture negative *S. pneumoniae* urine Ag positive PIV3	*S. pneumoniae* Parainfluenza virus 3 (Ct 31)	985 30	91.628 N/A
34	BAL	*P. aeruginosa* PCR not requested	*P. aeruginosa* Rhinovirus A (Ct 24)	52007 56	67.401 N/A
36	Sputum	Commensals	*E. corrodens* *N. mucosa* *V. parvula* *R. mucilaginosa* *P. melaninogenica* *P. jejuni* *N. meningitidis* *N. cinerea* *E. coli* *K. denitrificans* *A. defectiva* Influenza virus A (Ct 24)	195 164 39 37 31 21 20 13 12 7 7 2	32.939 27.703 6.588 6.25 5.236 3.547 3.378 2.196 2.027 1.182 1.182 N/A
37	Sputum	*C. freundii*	*C. freundii* Human gammaherpesvirus 4 Rhinovirus B (Ct25)	5660 44 15	70.119 N/A N/A
49	BAL	*P. aeruginosa* PCR not requested	*P. aeruginosa* SARSCov2 (Ct 19/21)	12016 3935	66.67 N/A
50	NTS	Negative	Influenza virus C	40	N/A

*H* high growth, *L* light growth, *S* scanty growth.

### Development of a data set to determine representative bacterial detection performance characteristics

Bacterial sensitivity and specificity were assessed at different abundance reporting thresholds (0.5%, 1%, 2%, 5% and 10%) at three sequencing time points (30 min, 2 h and 24 h) using ROC curves (Fig. 2 and Supplementary Data 10). At 1% abundance, sensitivity was 81% (30 min), 90% (2 h) and 94% (24 h), and specificity was 100% at all three time points apart from the 0.5% abundance threshold where 93% after 24 h, detecting sequencing an *S. aureus* at 0.8% of abundance (Table 6).

**Table 6:** Sensitivity and specificity for bacteria detection

Microbial abundance	30 min sequencing		2 h sequencing		24 h sequencing	
	Sensitivity	Specificity (%)	Sensitivity (%)	Specificity (%)	Sensitivity (%)	Specificity (%)
0.5%	81	100	90	93	94	93
1%	81	100	90	100	94	100
2%	81	100	87	100	90	100
5%	75	100	84	100	90	100
10%	70	100	80	100	88	100

There were three (3/33) false-negative sequencing results after 2 h at 1% abundance threshold. One with pure light growth of *B. cepacia* (sample 79) that was present by sequencing below 1% threshold at 2 h (but 3% after 24 h sequencing), one polymicrobial sample with scanty growth of *E. coli* (sample 73) and one poly-microbial sample with *E. meningoseptica* (sample 88) (Table 4). Conversely, sequencing identified *S. pneumoniae* in two samples from patients with community-acquired pneumonia that were not detected by culture (samples 51 and 20) but with a positive *S. pneumoniae* urinary antigen test and so were considered true positive findings (Table 4)

Significant additional detections were found in four BAL samples, including *Aspergillus fumigatus* and *P.jirovecii in* sample 73 both confirmed by qPCR. *Enterococcus faecium* with *vanA* was also detected in a sample with culture-reported vancomycin-resistant *E. faecium* (sample 83) (Supplementary Data 11).

### Identification of *Candida spp*

Different *Candida spp*. were cultured from five respiratory samples, reported as light or scanty growth (93, 73, 78, 36 and 94). Metagenomic sequencing correctly identified *Candida spp*. in 4 samples but missed *C. albicans* in a sample which yielded a scanty growth of *C. albicans* and in which 95% of the reads were classified as *S. aureus*. There were no false positive *Candida spp* reads in any sample above or below the reporting threshold (Supplementary Data 12).

### Prospective validation study on samples from patients with pneumonia

A prospective validation study was performed on 33 LRT samples using a 1% reporting threshold after 2 h sequencing. Results from routinely requested tests, including culture, targeted PCR, 16 S rRNA PCR or urinary pneumococcal Ag are compared with metagenomic sequencing reports in Supplementary Data 13.

Metagenomic results were concordant with results provided by the clinical laboratory for 60% of samples. In seven (21%) samples, additional potential pathogens were sequenced, and in 6 (18%) different results were provided by metagenomics and therefore classified as discordant (P9, P14, P20, P22, P24 and P27: Supplementary Data 13). Missed organisms comprised a metapneumovirus (Ct 32) in a pleural fluid containing 99.76% *S. pyogenes* reads (P9), and influenza virus A (Ct 27) in an NBL (P27). *C. freundii* in a sample also growing *P. aeruginosa* (P20) reported by metagenomics as *P. mirabilis* (80% reads) and *P. aeruginosa* (17% reads) and a CAP sample with moderate growth *P. aeruginosa* and mixed coliforms (P22) reported as *H. influenzae* (72% reads) with *P. aeruginosa* below the threshold at 2 h sequencing. The *H. influenzae* was considered the likely causative pathogen. Sample P10 had one read of influenza A virus in the 24 h sequence report only but not the 2 h report. There was one false positive result with *E. cloacae* reported by metagenomics in a culture-negative sample (P14).

*S. pyogenes* was sequenced in four culture-negative samples, confirmed by PCR in three samples, with the fourth sample taken from a patient with a previous culture-positive *S. pyogenes* respiratory sample a week before (Supplementary Data 13). Additional detections were reported after 2 h sequencing in nine samples. A metapneumovirus was detected in a sputum sample with *S. pyogenes* (sample P17) and bocaparvovirus in a BAL from a paediatric ICU with *P. jirovencii* detected by sequencing and confirmed by PCR (sample P21). In sample 175, *K. oxytoca* was additionally sequenced together with *E. cloacae* which was reported by routine culture (sample P13). The remaining additional detections were bacteria from three samples. These included *S. pneumoniae* or *H. influenzae* in three (P3, P7 and P23) each at >50% of total microbial reads, for which routine culture reported only commensals.

### Viral and bacterial genomic assembly using 24 h sequencing

Complete viral genome assembly was achieved in 17/36 of the viruses detected in true-positive samples in those used for the performance analysis, PCR for that particular virus yielded Ct values between 18 and 32. In 14/17 (42%) assembled viruses, the coverage at 10x was >90%. The highest Ct value for which this complete coverage was obtained was for a seasonal coronavirus 229E, present at Ct 32 (sample 47). Genomes were also assembled for two viruses identified by sequencing, both not detected by the routine targeted viral PCR panel: bocaparvovirus, in sample P21 and influenza virus C in sample 50. Influenza virus A and Influenza virus C were also assembled with good coverage of longer genome segments, but due to insufficient depth, assembly of the shortest two segments were not possible (Table 7).

**Table 7:** Viral genomes reference-based

Sample number	Virus	Ct value	Read count	Median read length	Mean read quality	Coverage %10×	Coverage %15×	Coverage %20×	Coverage %30×
1	SARSCov-2 (BF.5)	20/22	19,795	2252	13.3	99.736	99.659	99.622	99.244
6	PIV3	32	145	1994	14	88.695	54.178	29.673	10.697
11	SARSCov2 (BA.2.3)	20	2419	1430	13.5	99.348	99.161	98.9	95.459
16	RSV	26	1508	1828	14	97.996	97.339	96.636	91.814
17	SARSCov2 (BE.1)	22/20	16,074	1875	14	99.746	99.656	99.639	99.385
19	RSVB	25	267	2153	14.4	93.327	85.471	80.532	62.03
22	RSVB	25	703	2001	13.3	97.366	95.573	94.713	92.131
23	PIV4	27	289	1508	13	84.782	71.616	58.621	31.985
40	RSVA	24.5	199	1878	13	84.798	65.655	52.615	28.071
42	Human coronavirus 229E	32	733	1974	13.48	98.148	97.529	96.273	85.91
43	SARScov2(BA.5.2)	25/27	2207	1880	13.4	98.582	98.305	97.88	96.836
44	Adenovirus	19	55,546	1494	13.4	99.941	99.932	99.904	99.884
47	PIV3	34	92	2255	14.5	60.115	35.597	10.257	0
20	PIV3	31	318	3229	14.3	74.589	70.36	59.785	59.779
49	SASRCoV2 (BQ.1)	19/21	35,545	2190	13.8	99.973	99.659	99.656	99.615
18	CMV	N/A	60,731	2452	13.9	96.84	96.62	96.523	96.088
P21	Bocaparvovirus	20	7248	1771.5	12.7	95.81	95.18	94.89	93.65
50	FluC	N/A	1	385	11.8	0	0	0	0
			112	1745	12.2	92.558	90.825	89.767	80.761
			56	1464	12.3	79.941	73.043	68.853	46.763
			62	1629.5	12.9	87.769	83.372	77.462	66.377
			42	1625.5	12.6	77.183	76.700	72.697	60.396
			25	1312	11.6	71.998	59.048	19.480	0.000
			4	553.5	12.8	0	0	0	0
51	FluA	18	67	1398	12.3	92.00	87.74	83.60	71.23
			4	865	12.6	0.00	0.00	0.00	0.00
			28	1262	13.0	83.57	75.12	60.12	0.00
			34	1149	12.5	76.63	67.82	59.45	18.20
			2	688	13.0	0.00	0.00	0.00	0.00
			420	1613.5	12.9	94.04	92.88	90.77	89.03
			298	1537	13.2	93.98	93.76	92.31	87.14
			347	1515	13.0	100	97.86	95.90	91.85

More than 10,000 bacterial reads were obtained from 12/33 (33%) culture-positive samples in the training cohort, meeting the criteria for bacterial genome assembly of 12 bacteria. Five of the 12 bacteria had >90% coverage at 30× depth (Table 8).

**Table 8:** Bacterial genomes reference-based

Sample number	Organisms	Read count	Median read length	Mean read quality	Contigs	Largest contig	Total length	Reference length	Coverage	Coverage %10×	Coverage %15×	Coverage %20×	Coverage %30×
56	*S. pyogenes*	291547	3312	14.4	1	1.74	1.74	1.74	100.00	96.653	96.508	96.431	96.297
67	*K. pneumoniae*	10577	2115	13.52	7	5.34	5.69	5.71	99.65	5.778	4.479	3.666	2.509
69	*P. multocida*	18287	2861	14	1	2.28	2.28	2.46	92.68	87.619	73.309	48.401	9.682
70	*B. cenocepacia* (H)	314869	2337	12.7	3	3.58	7.75	7.76	99.87	69.46	63.936	59.357	51.308
72	S. marcescens (L)	569552	2764	13	2	5.05	5.08	5.04	100.79	85.431	84.932	84.481	83.548
78	*S. liquacences* (not reported)	244191	2199	13.5	3	5.29	5.3	5.59	94.81	N/A	N/A	N/A	N/A
85	*K.pneumoniae* (H)	186128	3558	13.4	7	5.25	5.59	5.83	95.88	91.058	90.904	90.41	88.317
87	*P. aeruginosa* (H)	367192	2203	13.5	1	6.25	6.25	6.54	95.57	81.615	78.298	75.473	70.637
90	*K. aerogenes* (H)	19182	2150	14	2	5.17	5.24	5.49	95.45	21.273	5.813	2.269	0.737
90	*E. coli* (not reported)	276158	2219	14	1	4.59	4.59	4.53	101.32	95.453	95.352	95.241	95.091
37	*C. freundii* (M)	57621	1516	13.5	2	4.91	5.04	5.6	90.00	70.843	40.812	18.852	3.746
49	*P. aeruginosa* (H)	115331	2786	13.5	1	6.23	6.23	7.05	88.37	N/A	N/A	N/A	N/A
P9	*S. pyogenes* (M)	542904	2050	14	1	1.74	1.74	1.74	100.00	95.67	95.623	95.586	95.532
P10	*E. hormaechei* (H)	114305	3994	14	2	4.71	4.71	5.04	93.45	89.15	88.978	88.734	87.544
P10	*S. aureus* (H)	107732	3383	15	1	2.81	2.81	2.86	98.25	95.768	95.741	95.722	95.454
P22	*H. influenzae* (not reported)	150369	2390	14	1	1.83	1.83	1.81	101.10	90.158	89.701	89.029	87.513
P20	*P. mirabilis* (not reported)	380754	1613	14	2	4.01	4.05	4.14	97.83	92.946	92.587	92.204	91.304
P23	*S. pneumoniae* (not reported)	40799	2330	13.64	2	2.1	2.1	1.8	116.67	83.052	77.169	67.421	49.274

Six additional genomes were recovered in the prospective validation study from 5 samples (P8, P10, P20, P22 and P23) comprising *S. pyogenes*, *E. hormaechei*, *S. aureus*, *H. influenzae*, *P. mirabilis* and *S. pneumoniae* (Table 8). There were 122 SNP differences between the two assembled RSV-B genomes and >100 SNP differences between the three parainfluenza virus 3 genomes. SNP differences between SARS-CoV-2 genomes were not compared because they were all different variants. There were 12,586 SNP differences between the two *S. pyogenes* genomes (P56 and P9).

### Operational factors and cost

Human DNA depletion steps were performed in a class I cabinet over 30 min (maximum 8 samples per run) before loading on an adjacent robot for a 30 min extraction run. Extracted microbial nucleic acid samples were then transferred to a separate molecular laboratory where RT, dsDNA synthesis and library preparation were performed over 90 min followed by PCR amplification over 150 min. Post PCR steps, including flowcell loading took 40 min, with first sequence reports produced after 30 min sequencing. In total the sample processing time through to 30 min sequence reporting was 7 h (Fig. 1). A second report was automatically generated at 2 h with sequencing continued for 24 h. Reagent costs for running 8 samples and loading all samples onto a single flow cell was £170 per sample.

## Discussion

This study presents a novel rapid pan-metagenomic protocol that can feasibly be used in a clinical laboratory to generate actionable reports within 7 h. Detailed data is presented on the impact of different method steps on human DNA depletion and preservation of microbial nucleic acid in samples with different combinations of natural or spiked organisms and from different sample types having variable background host and commensal DNA. Collectively, the data shows wide variation in the impact of method steps on the composition and recovery of respiratory pathogen sequences, given the significant intrinsic heterogeneity of clinical samples. Nevertheless, when the workflow was applied with pre-defined reporting thresholds to a prospective collection of pneumonia samples, comparing results with routine testing, it generated encouraging overall performance data. It also detected the most plausible respiratory pathogen where discrepant results were obtained with routine testing. On that basis, we conclude this prototype method has the potential to be taken forward for more detailed assessment as a clinically implementable test.

Overall, metagenomics showed concordance with routine testing for pathogen detection in 60% of cases. For bacteria, when discordance occurred in either the development or validation cohorts this was mostly due to metagenomics detecting a plausible dominant pathogen that failed to grow by culture likely due to prior antimicrobial treatment (*S. pyogenes*, *S. pneumoniae* and *H. influenzae*), especially significant were the *S. pyogenes* detections during the outbreak in 2022/23^20^. Metagenomics missed some bacteria of uncertain significance, identified mostly as light or scanty growth in polymicrobial cultures. A particularly informative example combining an additional and missed detection was when metagenomics reported the presence of *H. influenzae*, considered the likely cause of pneumonia, with *P. aeruginosa* below threshold in a patient with CAP, but culture reported moderate growth of *P. aeruginosa* and mixed coliforms. Such results would lead to different conclusions on the most likely causative pathogen and treatment options. It highlights the need to reassess what exactly is required or considered critical from a respiratory test: whether to identify pathogens most likely causing infection at that time versus knowing what future potential pathogens are present.

Another factor to consider alongside within-assay viral sensitivity levels is the additional benefit of identifying clinically significant RNA viruses (SARS-CoV-2, influenza and enterovirus) either in samples where viral PCR was not performed due to laboratory protocols (sputa), where testing was not requested by clinicians on BALs and in two cases by not being represented in the multiplex PCR panel (bocaparvovirus and influenza C virus). In one case, SARS-CoV-2 was identified in a patient with HAP not responding to antibiotics who had been negative twice by SARS-CoV-2 PCR during ten days prior to deterioration. The sample was de-anonymised after discussion with the clinical team according to ethics protocol, and the patient was started on steroids. These will all be important factors to consider in determining the clinical effectiveness and health-economic utility of incorporating a metagenomic test into the service offer.

Endonuclease treatment depletes free DNA and RNA both when artificially released from human cells during the method and naturally present from microbes in the sample at the time of collection, but should not affect nucleic acid in intact whole-cell microorganisms. This was supported by data that bacterial DNA in clinical samples decreased after endonuclease treatment but not when fresh bacteria were artificially spiked in clinical samples, as measured by the 16S Ct value (Supplementary Data 5 and 6). The digestion of free DNA and RNA by HL-SAN, in conjunction with the use of non-targeted primers, renders metagenomics somewhat less sensitive compared to targeted molecular methods like PCR, as it primarily detects intact microorganisms. Conversely, bacterial DNA increased again after the RT and dsDNA synthesis step, which may be due to RT activity on bacterial RNA (Supplementary Data 6). Previous studies have shown that one additional factor for missing detections could be due to the fact the organisms in the clinical samples are damaged during the storage time and temperature before being processed^21^. Other human DNA depletion methods able to sequence bacteria viruses divide the sample into two aliquots for different sample preparations, the supernatant for viral and the deposit for bacterial sequencing^8^ or detect only viruses or bacteria but both^10^. Those methods using the deposit, such as the saponin method, do not measure the amount of free DNA which is lost in the deposit after the centrifugation step^6,11^

This method is able to detect a broad range of different organisms with different compositions of the cell wall, different cell and genome sizes, using the same extraction and sequencing method for all of them in less than 24 h. This is why, depending on the organisms, the thresholds for reporting were differently established. For instance, thresholds for reporting culturable bacteria were established using the ROC curve analysis, and the minimum number of reads for reporting was set up using our previous experience using the same bioinformatic pipeline^12^. However, for organisms detected by target PCR in clinical laboratories, a more sensitive technique than culture and with smaller cell and genome size, the reporting thresholds could be set up to a smaller number of reads. A baseline threshold for RNA virus reporting was set as 1 read. This decision was supported by data generated from viral ROC curves, the absence of false positive results in any sample during the study and examples where only a single viral RNA read were reported in PCR-confirmed samples (samples 2 and 13). Furthermore, the default configurations of the Centrifuge score effectively minimise the likelihood of viral misclassification. This threshold provided encouraging sensitivity for RNA virus detection overall (94%) without compromising specificity. All RNA viruses were detected when present by PCR below a Ct of 30, although RNA viruses from both cohorts in samples with a Ct value > 30 were missed (parainfluenza virus, enterovirus, metapneumovirus and influenza A).

Another benefit of metagenomics beyond pathogen detection is to interrogate the genome for AMR or virulence factors and derive typing information for local outbreaks, and national surveillance of novel and unusual pathogens or even vaccine selection. Genome recovery was feasible for 42% of viruses and 33% of reported bacteria, an encouragingly high proportion from this heterogeneous clinical sample set. Influenza C and *VanA* gene detection were two notable examples here. Although full genome assembly for detailed SNP analysis^22^ is currently only attempted with 24 h read outputs, resistance genes are reportable after 2-h sequencing using this pipeline^12^ and inclusion of MLST schemes should also be feasible for 2 h reports^23,24^. Hence, a 30-min report can be generated for preliminary positive results, while a more comprehensive 2-h report can be prepared for detecting resistance genes and performing MLST analysis, all within the same day upon receiving the sample.

Serial dilution experiments identified RNA and DNA viruses at 70 copies/µL and bacteria or yeast at 10^3^ CFU/mL, although the latter varied by commensal bacterial load. Further work is required to determine formal LoDs including across a range of organisms, and assess against sensitivity required for clinical needs. Performance and thresholds for *Candida spp*., A*spergillus spp*., and other important organisms such as atypical respiratory pathogens and pathogens not sequenced in this study (*Mycobacteria* or *Rickettsia)* will require further work, although it is encouraging that some organisms not detected using the saponin-based method were identified here (*Chlamydia spp*, *Mycoplasma spp*, and *P. jirovecci*).

Finally, further adoption in a clinical laboratory setting will require automation to increase the number of specimens that can be currently processed by a single operator in a single run and by incorporating further controls. A negative control mock sample containing human cells rather than no-template control reported here to avoid missing low-level contamination that may not be detected when there is no carrier DNA in the sample. Additionally, a positive control for each run and an internal positive control organism added to each sample to ensure the method has completed satisfactorily in each sample tube^25,26^.

In conclusion, we have developed and clinically evaluated a prototype respiratory pan-metagenomic protocol suitable for further evaluation particularly aimed at routine microbiology laboratories to help progress the movement of metagenomic sequencing into a service setting^22^. It shows acceptable performance data, including when applied to a prospective cohort of adult and paediatric pneumonia patients with examples across the spectrum of causative pathogens. Further improvements from here will benefit from the evaluation of a broader range of sample types in different service laboratory settings to reach an agreement on defining an appropriate intended use, meeting the unmet clinical needs during the important first few hours of patient presentation. Assessment should consider all attributes an agnostic metagenomic test offers, including the value derived from detecting and characterising novel, unusual, unexpected or unrequested pathogens, examples of which are presented here and that replicate observations from our previous metagenomic studies^12^. We believe the method presented here provides a useful baseline, balancing the need to detect viruses, bacteria and fungi all in one sample, from which improvements can be considered.

### Supplementary information

Description of Additional Supplementary Files

Supplementary Data 1

Supplementary Data 2

Supplementary Data 3

Supplementary Data 4

Supplementary Data 5

Supplementary Data 6

Supplementary Data 7

Supplementary Data 8

Supplementary Data 9

Supplementary Data 10

Supplementary Data 11

Supplementary Data 12

Supplementary Data 13

Supplementary Data 14

Supplementary Data 15

reporting summary

## Data Availability

The data generated is available on ENA project number: PRJEB61294. Source data for Fig. 2 are available as Supplementary Data 10 and 11. The microorganisms sequenced on the negative control are available as Supplementary Data 15.

## References

[1] Chiu C. Y. & Miller S. A. Clinical metagenomics. *Nature Reviews Genetics*. (Nature Publishing Group, 2019). pp. 341–355. 10.1038/s41576-019-0113-7 PMC6858796 30918369

[2] The top 10 causes of death. https://www.who.int/news-room/fact-sheets/detail/the-top-10-causes-of-death#:~:text=The%20top%20global%20causes%20of,birth%20asphyxia%20and%20birth%20trauma%2C [cited 2024 Feb 5].

[3] Rudd, K. E. et al. Global, regional, and national sepsis incidence and mortality, 1990–2017: analysis for the Global Burden of Disease Study. *Lancet* **395**, 200–211 (2020). 31954465 10.1016/S0140-6736(19)32989-7 PMC6970225

[4] Cavallazzi, R. & Ramirez, J. A. How and when to manage respiratory infections out of hospital. *Eur. Respir. Rev.* **31**, 31 (2022). 10.1183/16000617.0092-2022 PMC9724804 36261157

[5] Bailey, A. L., Ledeboer N. & Burnham C. A. D. Clinical microbiology is growing up: the total laboratory automation revolution. *Clinical Chemistry*. (American Association for Clinical Chemistry Inc., 2019). vol 65, pp. 634–643. 10.1373/clinchem.2017.274522 30518664

[6] Shi, Y. Wang, G. Lau, H. C. & Yu, J. Metagenomic sequencing for microbial DNA in human samples: emerging technological advances. *Int. J. Mol. Sci.* **23**, 2181 (2022). 10.3390/ijms23042181 PMC8877284 35216302

[7] Thoendel, M. et al. Comparison of microbial DNA enrichment tools for metagenomic whole genome sequencing. *J. Microbiol. Methods* **127**, 141–145 (2016). 27237775 10.1016/j.mimet.2016.05.022 PMC5752108

[8] He, Y. et al. Enhanced DNA and RNA pathogen detection via metagenomic sequencing in patients with pneumonia. *J. Transl. Med.* **20**, 195 (2022). 35509078 10.1186/s12967-022-03397-5 PMC9066823

[9] Hasan, M. R. et al. Depletion of human DNA in spiked clinical specimens for improvement of sensitivity of pathogen detection by next-generation sequencing. *J. Clin. Microbiol.* **54**, 919–927 (2016). 26763966 10.1128/JCM.03050-15 PMC4809942

[10] Claro, I. M. et al. Rapid viral metagenomics using SMART-9N amplification and nanopore sequencing. *Wellcome Open Res.* **6**, 241 (2023). 37224315 10.12688/wellcomeopenres.17170.2 PMC10189296

[11] Charalampous, T. et al. Nanopore metagenomics enables rapid clinical diagnosis of bacterial lower respiratory infection. *Nat. Biotechnol.* **37**, 783–792 (2019). 31235920 10.1038/s41587-019-0156-5

[12] Charalampous, T. et al. Evaluating the potential for respiratory metagenomics to improve treatment of secondary infection and detection of nosocomial transmission on expanded COVID-19 intensive care units. *Genome Med.* **13**, 182 (2021). 34784976 10.1186/s13073-021-00991-y PMC8594956

[13] Fukumoto, H., Sato, Y., Hasegawa, H., Saeki, H. & Katano, H. Development of a new real-time PCR system for simultaneous detection of bacteria and fungi in pathological samples. *Int. J. Clin. Exp. Pathol.* **8**, 15479–15488 (2015). 26823918 PMC4713704

[14] Schell, W. A. et al. Evaluation of a digital microfluidic real-time PCR platform to detect DNA of Candida albicans in blood. *Eur. J. Clin. Microbiol. Infect. Dis.* **31**, 2237–2245 (2012). 22327343 10.1007/s10096-012-1561-6 PMC3939829

[15] Price, E. P. et al. Simultaneous identification of *Haemophilus influenzae* and *Haemophilus haemolyticus* using real-time PCR. *Fut. Microbiol.* **12**, 585–593 (2017). 10.2217/fmb-2016-0215 28604066

[16] Li, H. Minimap2: pairwise alignment for nucleotide sequences. *Bioinformatics* **34**, 3094–3100 (2018). 29750242 10.1093/bioinformatics/bty191 PMC6137996

[17] Kim, D., Song, L., Breitwieser, F. P. & Salzberg, S. L. Centrifuge: rapid and sensitive classification of metagenomic sequences. *Genome Res.* **26**, 1721–1729 (2016). 27852649 10.1101/gr.210641.116 PMC5131823

[18] Danecek, P. et al. Twelve years of SAMtools and BCFtools. *Gigascience* **10**, giab008 (2021). 33590861 10.1093/gigascience/giab008 PMC7931819

[19] Mikheenko, A., Saveliev, V. & Gurevich, A. MetaQUAST: evaluation of metagenome assemblies. *Bioinformatics* **32**, 1088–1090 (2016). 26614127 10.1093/bioinformatics/btv697

[20] Alcolea-Medina, A. et al. The ongoing *Streptococcus pyogenes* (Group A Streptococcus) outbreak in London, United Kingdom in December 2022: a molecular epidemiology study. *Clin. Microbiol. Infect.* **29**, 887–890 (2023). 36925107 10.1016/j.cmi.2023.03.001 PMC10769882

[21] Street, T. L. et al. Optimizing DNA extraction methods for nanopore sequencing of *Neisseria gonorrhoeae* directly from urine samples. *J. Clin. Microbiol.* **58**, e01822–19 (2020). 31852766 10.1128/JCM.01822-19 PMC7041563

[22] Edgeworth, J. D. Respiratory metagenomics: route to routine service. *Curr. Opin. Infect. Dis.* **36**, 115–123 (2023 Apr). 36853748 10.1097/QCO.0000000000000909 PMC10004755

[23] Taxt, A. M., Avershina, E., Frye, S. A., Naseer, U. & Ahmad, R. Rapid identification of pathogens, antibiotic resistance genes and plasmids in blood cultures by nanopore sequencing. *Sci. Rep.* **10**, 7622 (2020). 32376847 10.1038/s41598-020-64616-x PMC7203151

[24] Page, A. J. & Keane, J. A. Rapid multi-locus sequence typing direct from uncorrected long reads using *Krocus*. *PeerJ* **6**, e5233 (2018). 30083440 10.7717/peerj.5233 PMC6074768

[25] López-Labrador, F. X. et al. Recommendations for the introduction of metagenomic high-throughput sequencing in clinical virology, part I: wet lab procedure. *J. Clin. Virol.* **134**, 104691 (2021). 33278791 10.1016/j.jcv.2020.104691

[26] Atkinson, L. et al. Untargeted metagenomics protocol for the diagnosis of infection from CSF and tissue from sterile sites. *Heliyon* **9**, e19854 (2023). 37809666 10.1016/j.heliyon.2023.e19854 PMC10559231

