# Publisher Correction: Unified metagenomic method for rapid detection of microorganisms in clinical samples

**DOI:** 10.1038/s43856-024-00579-8

**Published:** 2024-07-26

**Authors:** Adela Alcolea-Medina, Christopher Alder, Luke B. Snell, Themoula Charalampous, Alp Aydin, Gaia Nebbia, Tom Williams, Simon Goldenberg, Sam Douthwaite, Rahul Batra, Penelope R. Cliff, Hannah Mischo, Stuart Neil, Mark Wilks, Jonathan D. Edgeworth

**Affiliations:** 1 Infection Sciences, Synnovis, London, UK; 2 Center for Clinical Infection and Diagnostics Research, Guys’ and St. Thomas’ NHS Foundation Trust, London, UK; 3 Department of Infectious Diseases, King’s College London, London, UK; 4 Quadram Institute Bioscience, Norwich, UK; 5 Department of Infectious Diseases, Guys’ and St Thomas’ NHS Foundation Trust, London, UK; 6 Queen Mary University of London, London, UK

**Keywords:** Clinical microbiology, Metagenomics

Correction to: *Communications Medicine* 10.1038/s43856-024-00554-3, published online 07 July 2024

In this article, the affiliation details for Author Gaia Nebbia were incorrectly given as ‘Quadram Institute Bioscience, Norwich, UK’ but should have been ‘Department of Infectious Diseases, Guys’ and St Thomas ‘NHS Foundation Trust, London, UK.’. The original article has been corrected.

