# Supplementary material for: Unified metagenomic method for rapid detection of microorganisms in clinical samples
Source: Commun Med (Lond). 2024 Jul 7;4:135. doi: 10.1038/s43856-024-00554-3 (PMC11228040; doi:10.1038/s43856-024-00554-3)
Supplement: Supplementary file 1 — Description of Additional Supplementary Files [file 43856_2024_554_MOESM1_ESM.pdf]

Description of Additional Supplementary Files

File name: Supplementary Data 1.

File Description: Viruses considered pathogenic.

File name: Supplementary Data 2.

File Description: URTS depletion.

File name: Supplementary Data 3.

File Description: LRT depletion.

File name: Supplementary Data 4.

File Description: Viral loss.

File name: Supplementary Data 5.

File Description: Bacteria loss.

File name: Supplementary Data 6.

File Description: RNA recovery.

File name: Supplementary Data 7.

File Description: Viral LoD

File name: Supplementary Data 8.

File Description: Bacteria LoD

File name: Supplementary Data 9.

File Description: Candida LoD

File name: Supplementary Data 10.

File Description: Viral performance.

File name: Supplementary Data 11.

File Description: Bacteria performance.

File name: Supplementary Data 12.

File Description: Candida performance.

File name: Supplementary Data 13.

File Description: Prospective samples.

File name: Supplementary Data 14

File Description: Primers

File name: Supplementary Data 15

File Description: Negative controls.
